# Long-term treatment with transcranial pulsed electromagnetic fields improves movement speed and elevates cerebrospinal erythropoietin in Parkinson’s disease

**DOI:** 10.1371/journal.pone.0248800

**Published:** 2021-04-28

**Authors:** Bente Rona Jensen, Anne Sofie Bøgh Malling, Sissel Ida Schmidt, Morten Meyer, Bo Mohr Morberg, Lene Wermuth

**Affiliations:** 1 Department of Neurology, Odense University Hospital, University of Southern Denmark, Odense, Denmark; 2 Department of Clinical Research/BRIDGE, University of Southern Denmark, Odense, Denmark; 3 Department of Neurobiology Research, Institute of Molecular Medicine, University of Southern Denmark, Odense, Denmark; Emory University, UNITED STATES

## Abstract

**Background:**

Parkinson’s disease is characterized by motor dysfunctions including bradykinesia. In a recent study, eight weeks of daily transcranial stimulation with bipolar pulsed electromagnetic fields improved functional rate of force development and decreased inter-hand tremor coherence in patients with mild Parkinson’s disease.

**Objective:**

To investigate the effect of long-term treatment with transcranial bipolar pulsed electromagnetic fields on motor performance in terms of movement speed and on neurotrophic and angiogenic factors.

**Methods:**

Patients diagnosed with idiopathic Parkinson’s disease had either daily 30-min treatment with bipolar (±50 V) transcranial pulsed electromagnetic stimulation (squared pulses, 3ms duration) for three eight-week periods separated by one-week pauses (T-PEMF group) (n = 16) or were included in a PD-control group (n = 8). Movement speed was assessed in a six-cycle sit-to-stand task performed on a force plate. Cerebrospinal fluid and venous blood were collected and analyzed for erythropoietin and vascular endothelial growth factor.

**Results:**

Major significant improvement of movement speed compared to the natural development of the disease was found (p = 0.001). Thus, task completion time decreased gradually during the treatment period from 10.10s to 8.23s (p<0.001). The untreated PD-control group did not change (p = 0.458). The treated group did not differ statistically from that of a healthy age matched reference group at completion of treatment. Erythropoietin concentration in the cerebrospinal fluid also increased significantly in the treated group (p = 0.012).

**Conclusion:**

Long-term treatment with transcranial bipolar pulsed electromagnetic fields increased movement speed markedly and elevated erythropoietin levels. We hypothesize that treatment with transcranial bipolar pulsed electromagnetic fields improved functional performance by increasing dopamine levels in the brain, possibly through erythropoietin induced neural repair and/or protection of dopaminergic neurons.

## Introduction

Parkinson’s disease (PD) is a progressive chronic disorder characterized by motor dysfunctions including bradykinesia, rigidity, akinesia, tremor, and postural instability. Bradykinesia, i.e. slowness of movements, was perceived as the most prevalent troublesome symptom by patients with early PD (< 6 years) [1]. The ability to perform fast muscle contractions is impaired in PD even though no deficits in maximal force occur [2, 3]. Coordination and balance are also impaired in PD [4, 5]. These specific motor deficits contribute to the slow movement speed in patients with PD, which constitutes a challenge for daily activities. The primary pathological processes involve degeneration of nigrostriatal dopaminergic neurons leading to reduced striatal dopamine. Dopamine is the major catecholamine neurotransmitter in the brain and is highly significant for motor control processes [6–8].

Medical treatment of PD is purely symptomatic as no cure exists. PD has traditionally been considered a single-region disease in the basal ganglia but has recently been proposed as a system-level disorder that includes functional interactions between brain regions such as the dopamine dependent motor circuit (cortico-striatal-thalamic circuit) and the cognitive circuit (frontal-striatal circuit) [9]. This has stimulated new research directed towards further understanding of the disease, its functional consequences, and innovative treatments.

Electromagnetic stimulation of biological tissue is a non-invasive rapidly emerging technique. The biophysical effect of pulsed electromagnetic stimulation is to induce ion currents in the tissue and to depolarize the membrane potential slightly [10]. Electromagnetic stimulation in animals and *in-vitro* seems to enhance cellular activity and stimulate growth-related responses and regeneration [10]. Thus, pulsed electromagnetic fields (PEMF) stimulated nerve growth and attenuated nerve abnormalities, increased microvascular blood flow and tissue oxygenation, and increased capillary density [11–14]. Treatment with PEMF may thus delay disease progression or even induce neuro-repair in PD.

In a recent clinical trial, we applied transcranial PEMF (T-PEMF) in patients with idiopathic PD [15–17]. This study focused on non-invasive functional effect measures and was the first to apply T-PEMF in PD. We found that 30-min daily treatment with T-PEMF over eight weeks were superior to placebo treatment to increase functional rate of force development during a cyclic chair rise task, and that inter-hand coherence was reduced in the least affected patients with PD [15, 17]. However, structural changes in the brain take time and eight weeks of treatment is unlikely to be sufficient. Thus, even longer treatment periods are needed to better understand the potential of this treatment modality [15, 17].

We speculate on a potential connection between T-PEMF treatment and cerebral levels of erythropoietin (EPO), vascular endothelial growth factor (VEGF), and dopamine. EPO is present in the central nervous system, has a multimodal neuroprotective profile and has a key role in neuroprotection and neuro repair [18, 19]. EPO releasing cells such as Er-NPC promoted neural repair and recovery of function in a mouse model of spinal cord traumatic injury, and EPO improved memory and spatial learning in a rat model of Alzheimer’s disease [20, 21]. Signs of neuroprotective effects of EPO were also found in an animal model of PD [22, 23]. VEGF mediates angiogenesis, neural migration and neuroprotection [24, 25] and, along with EPO may be a potential therapy for certain neurological disorders. The literature mainly comprises animal and *in-vitro* studies, however, and our knowledge in humans is limited.

The present study, aimed to test the hypothesis that long-term treatment with T-PEMF would improve motor performance (in terms of movement speed) and stimulate production of neuroprotective and angiogenetic compounds (EPO and VEGF) in the brain in patients with PD.

## Materials and methods

### Study design

The study was a prospective interventional treatment study with parallel group design and partially blinded outcome assessment. Treatment outcomes were compared to natural development of the disease and to healthy controls. The study was approved by the Regional Scientific Ethical Committees for Southern Denmark (S-20160106) and the Danish Medicines Agency (CIV-16-02-014744) and was conducted in accordance with the Declaration of Helsinki. All participants gave written informed consent prior to participation.

### Participants

Patients diagnosed with idiopathic PD, according to the UK Brain Bank criteria, were recruited from the clinic. Inclusion criteria were a Mini-Mental State Examination (MMSE) score >22, age >18 years, ability to give written informed consent, and to obtain certification in the use of the T-PEMF device. Exclusion criteria were any known neuromuscular or neurological diseases other than PD that might interfere with motor function; psychopathological treatment of other conditions than depression; substance abuse; active medical implants; pregnancy; current or previous cancer in the head, brain, or neck region; leukemia; autoimmune disease; epilepsy; and open scalp wounds. Treatment compliance >80% was required to be included in the analyses. Of the 24 patients recruited, 16 patients were included in an active T-PEMF treatment group and eight patients were included in a PD-control group (no T-PEMF treatment). The PD-control group was included in the study to indicate the time course of the typical development of PD. The patients were pharmacologically well-treated, and the medication remained unchanged for at least six weeks prior to and throughout the intervention period (T-PEMF group). Disease severity was assessed using the Unified Parkinson Disease Rating Scale (UPDRS) and used to describe the study population [26]. In addition, healthy reference values (n = 39) based on data from an age and sex matched healthy cohort were extracted without any knowledge of their other characteristics [27].

Eight participants from the active group in the clinical trial (NCT02125032) (8 weeks treatment) were enrolled in the present study (3x8 weeks treatment) (T-PEMF group: n = 6, PD-control group: n = 2). The eight participants from the previous clinical trial were recruited without any knowledge regarding prior results from the clinical trial. The distribution of the eight participants between the T-PEMF group and the PD-control group was by convenience. The average time from last treatment in the 8-week intervention to enrolment in the present long-term intervention was 25 months (range 14–32 months), so no carry over effect from the 8-week intervention was expected. No results presented in the present study have been reported previously.

### Treatment intervention

In the intervention group, daily T-PEMF was applied for three eight-week periods, separated by one-week pauses. Treatment with T-PEMF has not been conducted for such a long period previously, and the one-week pause between treatments periods were required by the Danish Medicines Agency to permit the study. Thus, the total treatment intervention period was 26 weeks. The treatment was home-based, and each daily session lasted 30 min. T-PEMF was applied through seven coils placed in a helmet-like shape, with one coil in the central occipital region, one in each frontal-parietal region, and two bilaterally in the temporal region (anterior and posterior) (Re5 NTS Parkinson Treatment System, Re5, Frederiksberg, Denmark). The seven coils were connected to an external pulse generator that generated bipolar, squared pulses (±50 V, 3 ms, 50 Hz) to initiate rapid current changes in the coils, giving rise to rapidly changing, time-dependent electromagnetic fields. An electromagnetic field penetrates through electrically insulated tissue (e.g. the skull) and induces a driving force on charged particles and thereby electrical currents in the brain. The peak E-field intensity has been estimated to approximately 2.5 mV/cm near the coil [10], and the spatial distribution is highly dependent on the steep gradient of the applied bipolar pulsed electromagnetic field.

Treatment initiation and duration were controlled by a pre-programmed chip-card inserted into the pulse generator. Date and time of each treatment were stored on the chip card and used to assess treatment compliance. The participants were in contact with the investigators once a week (telephone or e-mail). They were also given a hotline telephone number so they could contact the investigators at any time if they experienced adverse effects or had treatment-related questions.

### Effect measures

#### Movement speed

The primary outcome was movement speed, which was assessed in a six-cycle sit-to-stand (STS) task as described previously [15, 28]. In short, the participants sat on a chair with their arms folded at chest height. Seat height was 120% tibia length. The knee angle was 90–100 degrees and the feet were placed on a force plate (AMTI, 120x90 cm, BP6001200, USA) with an inter-feet distance corresponding to shoulder width. The criteria for an approved trial were fully extended knees in the standing position and back-to-backrest contact in the sitting position. Familiarization was performed before measurements. The researchers performing the examinations (week 17 and week 27) were blinded to prior results of the STS task.

The participants were asked to perform the task as fast as possible. Three approved STS trials were performed with rest between trials to avoid fatigue. If the last trial was the fastest an additional trial was performed to ensure maximal performance. Force plate data were sampled (1000Hz), A/D converted (16-bit, Data Translation Inc., USA) and filtered with a zero phase-lag, second order 25 Hz low-pass Butterworth filter. The average of the data from the two fastest approved trials is reported. The STS-task was performed three times: just before initiation of the treatment, within one day after completion of the second treatment period and within one day after completion of the third treatment period. The measurements were performed with the patients in self-reported “on”-state and at approximately the same time of the day.

STS completion time was calculated as the time from the first vertical ground reaction force peak of the 1st repetition to the 1st vertical ground reaction force peak of the 6th repetition, thus reflecting the time for five repetitions. Functional rate of force development (RFD_STS-up_) was calculated as the average slope of the increasing force of the 1st force peak and decrease (RFD_STS-down_) was the decreasing force of the 2^nd^ force peak in the 2^nd^-6^th^ repetition, within the time interval corresponding to 30–70% of the exerted peak force. Results are reported in bodyweight per second (BW/s). The exact time while sitting and standing was not measured, and we used instead the time interval from the first to the second force peak and from the second to the first force peak, to largely represent the standing and the sitting time, respectively.

#### Cerebrospinal fluid (CSF) and blood samples

Eight patients from the T-PEMF group underwent lumbar puncture just before treatment and within one day after treatment completion. The patients were consecutively recruited while excluding patients taking anticoagulants. Lumbar puncture was performed using a 22-gauge spinal needle. CSF (5 ml) was collected with polypropylene syringes in an intervertebral lumbar space and subsequently stored within 30-min at—80°C. Venous blood samples were collected from 13 patients (eight in the T-PEMF group, five in the PD-control group) before treatment and within one day after the treatment completion. The patients were consecutively recruited (convenience sub-sample).

Blood samples were collected from the antecubital vein and centrifuged at 2000g for 10 min. The supernatants were stored in CRYO-S tubes and stored within 30-min at -80°C. CSF and blood samples were analyzed for EPO and VEGF using the MSD U-PLEX Human EPO Assay (#K151VXK) / V-PLEX Angiogenesis Panel 1 Human Kit (#K15190D) (Mesoscale Discovery, Rockville, MD, USA) and a SECTOR Imager 6000 (Mesoscale Discovery) plate reader according to the manufacturer’s instructions. Data were analyzed using MSD Discovery Workbench software. Analyses were performed in duplicate and reported as average values. The laboratory technician was blinded to sample identity.

### Statistical analysis

Statistics were performed in SAS 9.4 (USA). Normality tests were performed (Shapiro-Wilk). A linear mixed model with parallel group approach was used to assess treatment effects on STS performance between PD groups (T-PEMF group vs PD-control group). The model used was: *Performance outcome = treatment time treatment×time*, performed with unstructured covariance. Dependent performance outcome; STS completion time, RFD_STS-up_, RFD_STS-down_, standing time and sitting time. Independent factors; time (week 0, week 17 and week 27) and treatment (T-PEMF and no T-PEMF). Unstructured covariance was used to allow inhomogeneous variance across the treatment period. For hypothesis test a significance level of alpha = 0.05 was used. Post hoc paired two-sample t-test (two-tailed) between time points (week 0 versus week 27) was performed for STS performance means to assess differences from baseline to post-treatment in the two PD groups. Significant values were corrected for multiple testing according to Bonferroni.

To analyze the effect of T-PEMF treatment (T-PEMF group) on CSF-EPO and CSF-VEGF a two-sample T-test (week 0 versus week 27) was used (two-tailed). To assess the effect of T-PEMF treatment (T-PEMF group versus PD-control group) on plasma-EPO and plasma-VEGF a repeated measure ANOVA was used.

Comparisons between the T-PEMF group and the healthy reference group at specific time points were performed using unpaired two-sample t-test (two-tailed).

Baseline comparisons between PD groups on participant characteristics were performed by chi-square (sex), Mann-Whitney U-test (Mini-Mental State Examination Score and Hoehn and Yahr Score, not normally distributed) and unpaired two-sample t-test (age, height, body weight, UPDRS, Levodopa equivalent dose, disease duration).

Finally, correlations between UPDRS score at baseline and absolute as well as relative change in STS completion time were calculated (Pearson).

Statistics are reported in accordance with general guidance on statistical reporting (SAMPL guidelines).

## Results

### Participants

In the T-PEMF group, one patient withdrew, and one was excluded from the study due to medical conditions unrelated to the T-PEMF treatment. Baseline characteristics for the PD groups are shown in Table 1. We found no significant between-group differences in age, height, bodyweight, UPDRS-total, UPDRS-motor, UPDRS-bradykinesia, MMSE score, levodopa equivalent dose or disease duration. Average treatment compliance across the intervention period (3x8 weeks) was 97.6% (SD 2.8) and no participants were excluded due to low compliance. The healthy reference group had an age of 66.8 years (SD 6.5), bodyweight of 76.6 kg (SD10.8) and height of 1.74 m (SD 0.09).

**Table 1:** Characteristics of the participants with Parkinson’s disease (PD) in the T-PEMF intervention group and the PD-control group.

	T-PEMF, N = 14	PD-Control, N = 8	*P*-values
Sex (female/male)	5/9	2/6	0.604^a^
Age (years)	66.6 (7.6)	70.5 (6.3)	0.240^b^
Height (cm)	172 (8.7)	172 (6.6)	0.925^b^
Weight (kg)	73.5 (11.4)	73.3 (10.5)	0.960^b^
UPDRS-total	46.4 (22.2)	35.3 (5.1)	0.099^c^
UPDRS-motor	27.8 (15.0)	21.1 (3.4)	0.137^c^
UPDRS-bradykinesia	14.6 (8.1)	12.3 (3.2)	0.351^c^
Hoehn-Yahr score	2.0 (2.0–2.9)	2.3 (2.0–2.5)	0.904 ^d^
MMSE	29.5 (28.0–30.0)	30.0 (28.8–30.0)	0.527^d^
Levodopa equivalent dose (mg/day)	498.3 (259.1)	469.4 (148.6)	0.777^b^
Disease duration (years)	6.2 (3.5)	5.3 (3.7)	0.581^b^

^a^ Chi-square test

^b^ T-test, equal variance

^c^ T-test, unequal variance (Cochran-Cox)

^d^ Mann-Whitney U-test

Unified Parkinson’s disease Rating Scale total score (UPDRS-total, range 0–199), motor score (UPDRS-motor, range 0–108) and bradykinesia score (UPDRS-bradykinesia, range 0–36). Mini-Mental State Examination (MMSE, range 0–30).

Disease duration is the time period from diagnosis to study inclusion. Data are means (SD), except for MMSE and Hoehn-Yahr where medians and inter-quartile ranges are reported.

### Sit-to-stand/movement speed

**Fig 1:**
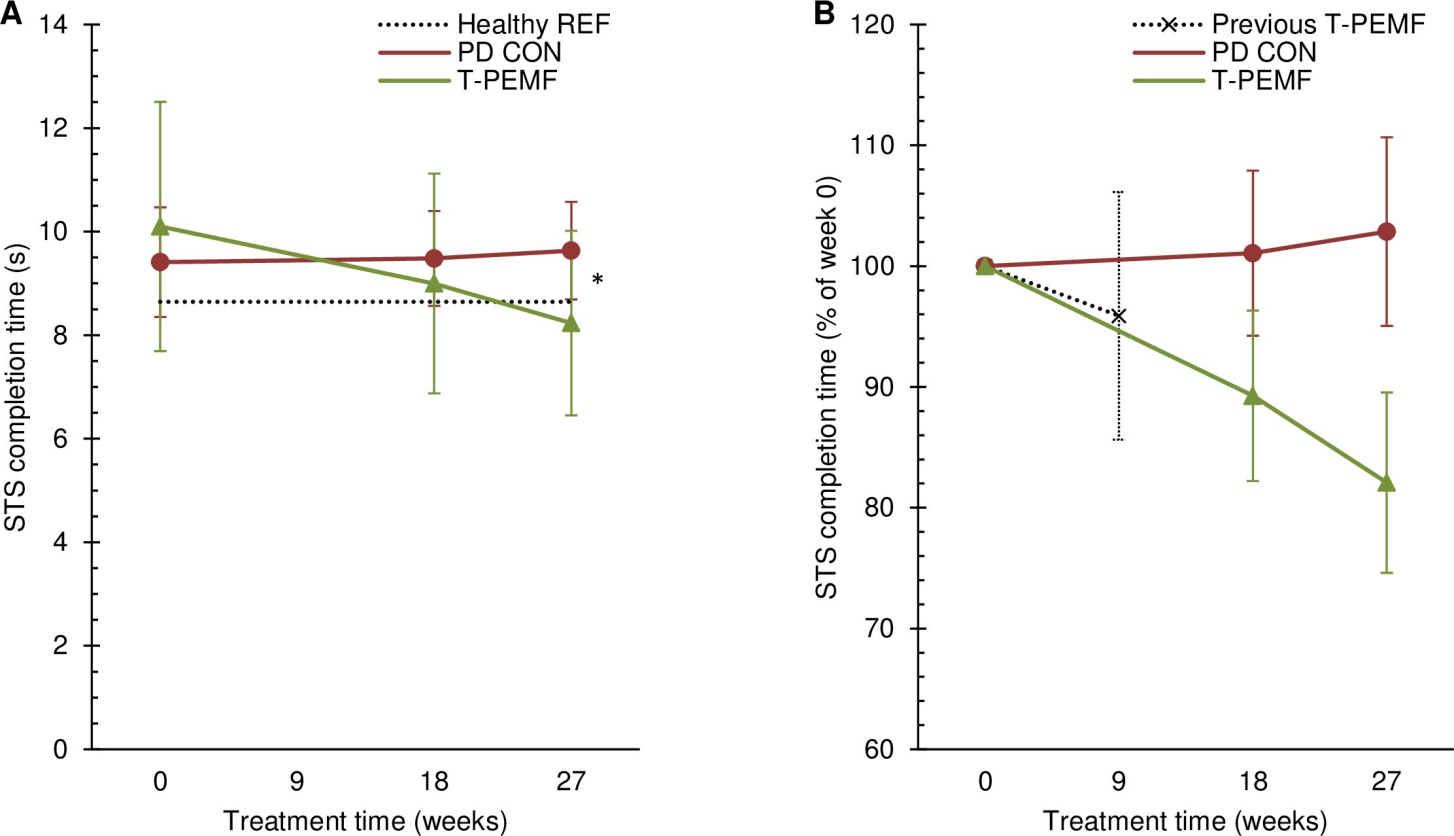
Sit-to-stand completion time. Absolute (A) and relative (B) completion time over the study period for the group receiving T-PEMF treatment (T-PEMF; green, solid line with triangles) and the PD control group (PD CON; red, solid line with circles). A: * Significantly different performance course over time (*P*<0.05) between T-PEMF and PD CON. Healthy reference level is indicated (Healthy REF; grey, dotted line). B: The relative change in a T-PEMF group from our previous study by Malling et al. after 8 weeks of treatment is indicated (Previous T-PEMF; black, dotted line with x’s) [15].

The time course of the STS completion time differed significantly between the T-PEMF group and the PD-control group (p = 0.001). The T-PEMF group completed the STS-task in 10.10s (SD 2.41) at week 0 and decreased significantly to 8.23s (SD 1.78) post treatment (p<0.001) (Fig 1A). The PD-control group did not change significantly across time (p = 0.458). The healthy reference group completed the STS-task in 8.64s (SD 1.61). Completion time was significantly longer for the PD group than for the healthy reference group at baseline (p = 0.015). However, no between group (T-PEMF group vs. healthy reference group) difference in completion time was found post treatment (p = 0.429). Relative changes for the two PD groups are shown in Fig 1B. The relative difference between the two PD groups increased gradually across the intervention period and was 21% points at endpoint. Data from our recent eight-week T-PEMF treatment study has been added to Fig 1B for comparison [15].

**Fig 2:**
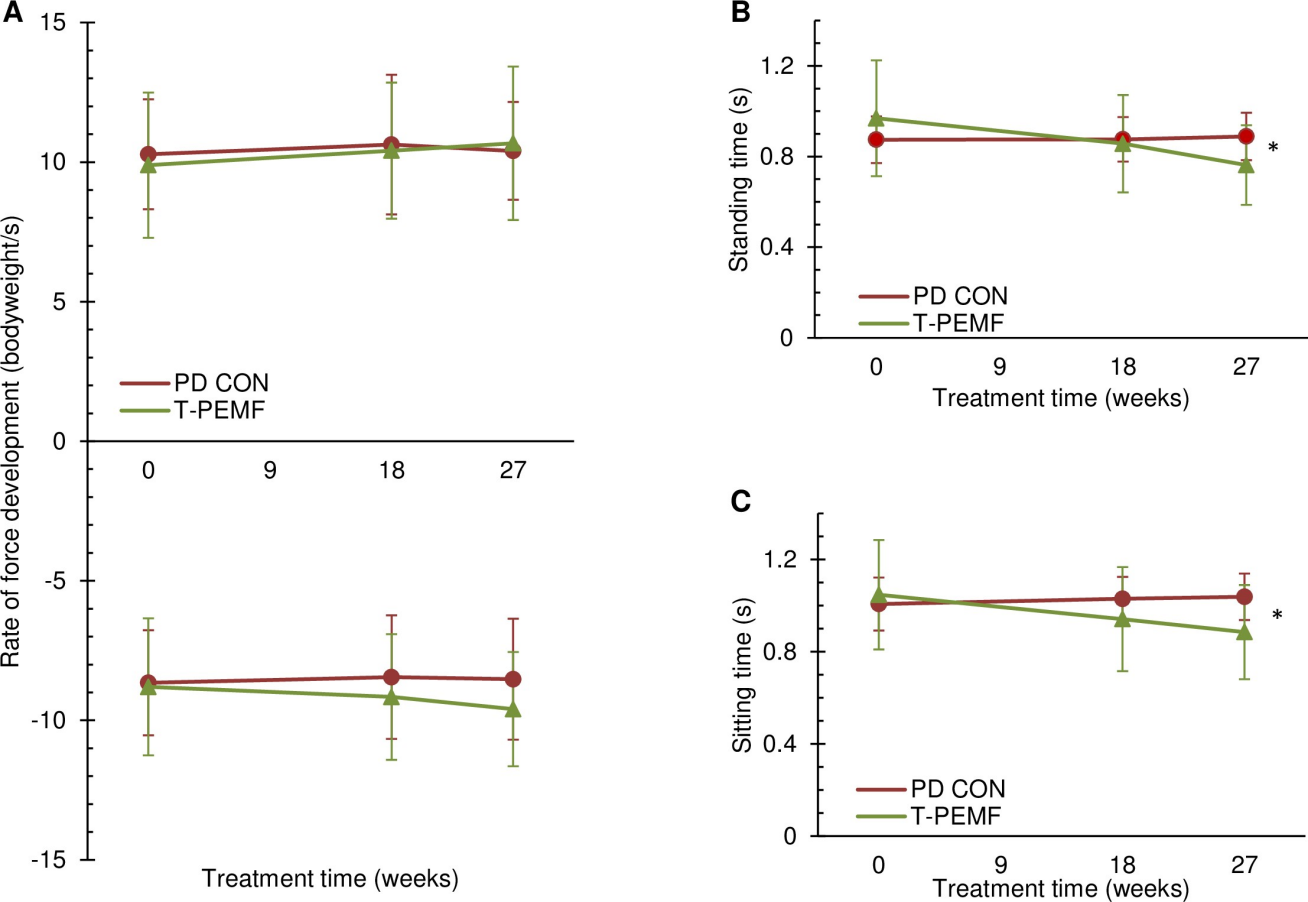
Subdivision of the sit-to-stand movement. Rate of force development during rising from a chair (A, upper) and lowering to a chair (A, lower), standing time (B) and sitting time (C) for the group receiving T-PEMF treatment (T-PEMF; green, solid line with triangles) and the PD control group (PD CON; red, solid line with circles). * Significant between-group difference in performance over time (*P*<0.05).

**Fig 3:**
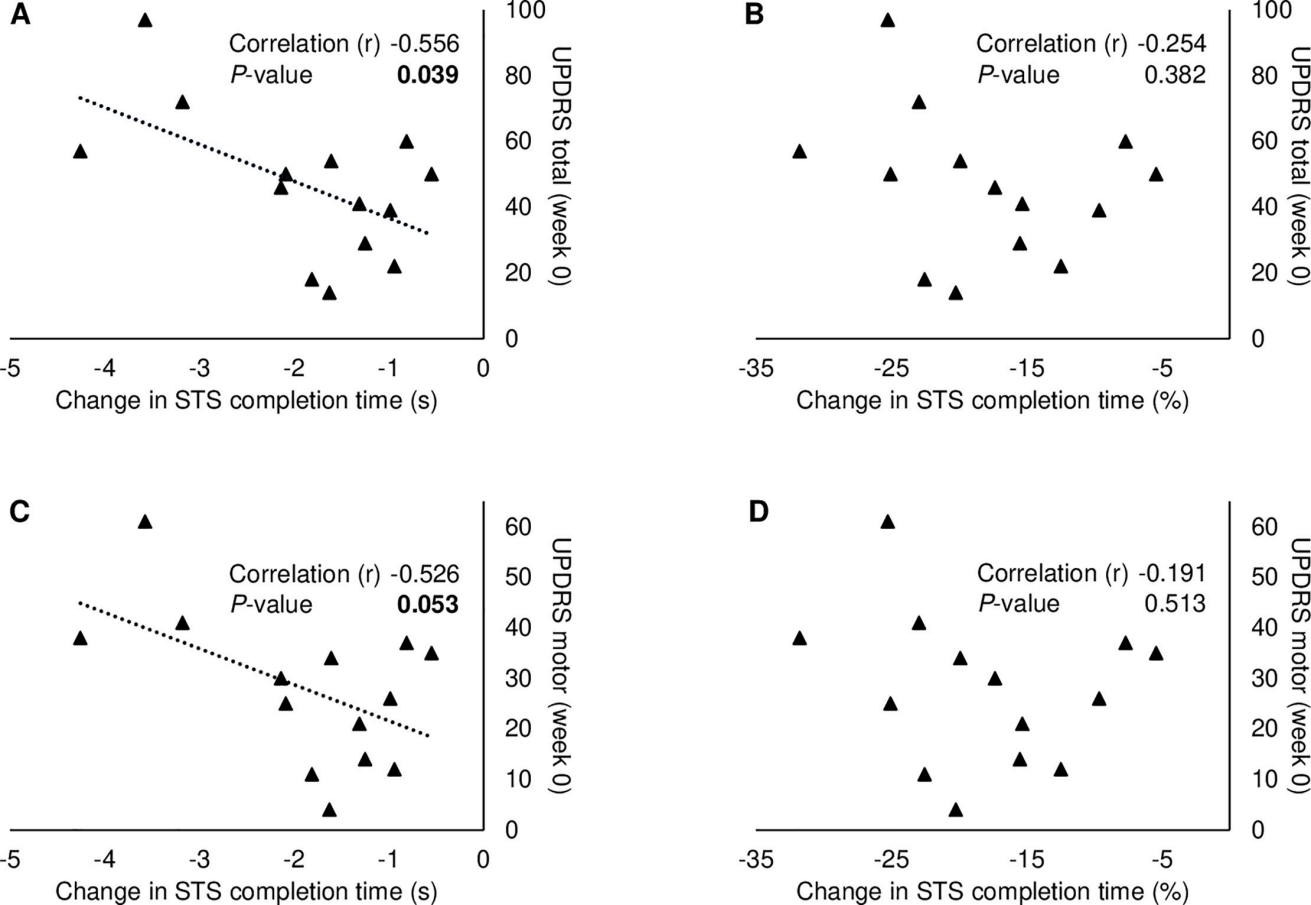
Association between disease severity and change in sit-to-stand completion time from week 0 to week 27 in the T-PEMF group. Pearson correlations between UPDRS total score and the absolute (A) and relative (B) change of completion time, and between UPDRS motor score and the absolute (C) and relative (D) change of completion time.

Subdivision of the STS completion time showed no between-group differences regarding rate of force development although a non-significant trend was seen (Fig 2A). However, the time periods largely representing standing and sitting time, respectively, were significant shorter in the T-PEMF group (Fig 2B and 2C). A significant moderate negative correlation was found between absolute changes in STS completion time and UPDRS-total score in the T-PEMF group and a tendency was found for the UPDRS-motor score (Fig 3A and 3C) [29]. No association was found between UPDRS scores and the relative change of completion time (Fig 3B and 3D).

### Biomarkers

**Fig 4:**
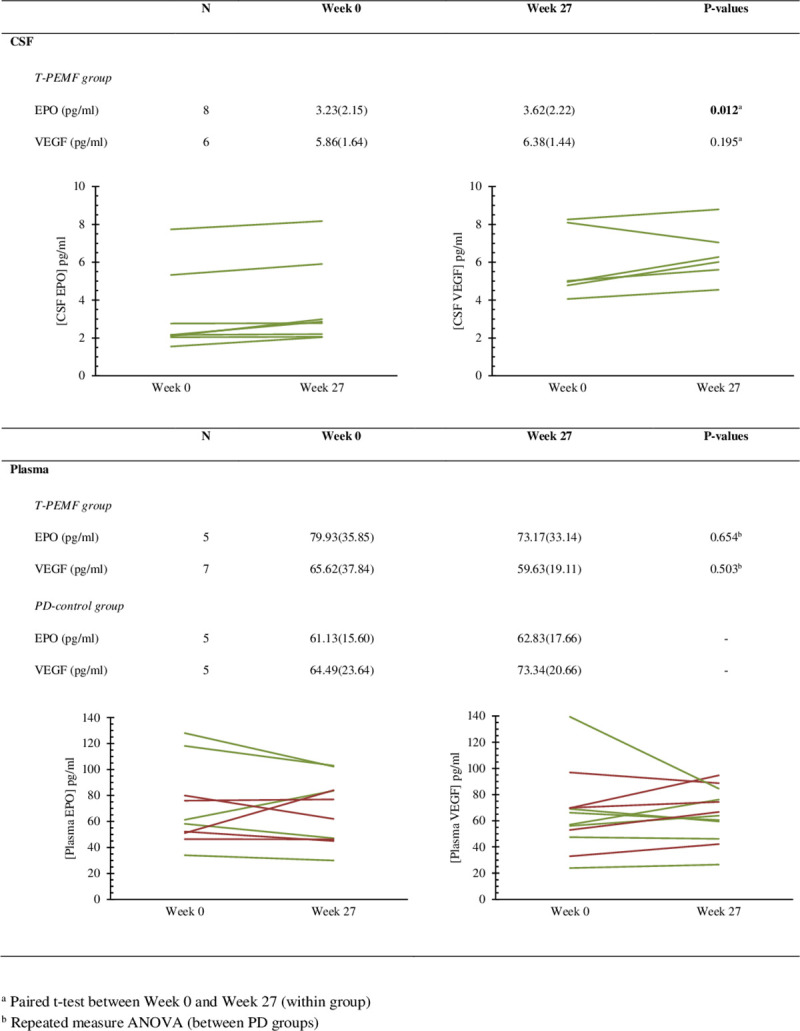
Erythropoietin (EPO) and vascular endothelial growth factor (VEGF). Group mean (SD) of erythropoietin and vascular endothelial growth factor concentration in cerebrospinal fluid (CSF) and plasma in patients with Parkinson’s disease who were either treated with transcranial pulsed electromagnetic field treatment (T-PEMF, green lines) or were in the PD-control group (red lines). Individual values are displayed.

EPO and VEGF concentrations in CSF and in venous blood plasma are presented in Fig 4. CSF-EPO concentrations increased significantly in response to the T-PEMF treatment intervention (all patients increased (n = 8)). CSF-VEGF concentration increased in five out of six patients included in the analysis (non-significant).

## Discussion

The present study is the first to document that T-PEMF treatment applied for three eight-week periods improves movement speed significantly when compared with the untreated PD control group. Thus, the characteristic slowness of movements was markedly reduced. Moreover, enhanced levels of CSF-EPO in response to the treatment intervention were found. A possible causal mechanistic link between T-PEMF, CSF-EPO and movement speed are suggested.

Several animal models have shown a neuroprotective action of EPO in neurodegenerative diseases when injected intravenously or infused directly into the brain. [e.g. 22, 30]. When systemic (intravenous) administration of EPO and intrastriatal injected EPO were compared in a PD animal model, the blood-brain barrier appeared to limit the passage of EPO into the brain as only low levels of EPO crossed despite a high intravenous dose [18, 31, 32]. Systemic administration of EPO in patients with PD is thus unlikely to be appropriate. Nevertheless, improved autonomic function and cognition were found in patients with PD when assessed clinically with the Non-Motor Symptom Scale after five weeks of intravenous EPO administration twice a week [33]. Infusion of EPO or neuroprotective EPO-like compounds directly into the human brain is a theoretical possibility, but its invasive character and expected inhomogeneous spatial distribution in brain tissue make this less realistic. To overcome these challenges, treatments that upregulate the intrinsic production of EPO in the brain would be preferable. We found that long-term treatment of the brain with T-PEMF increased CSF levels of EPO, indicating enhanced levels of locally produced intracerebral EPO. An animal model of PD showed that intrastriatal administered EPO has specifically protected dopaminergic neurons from cell death and improved motor performance [34] and thus, we cautiously hypothesize that T-PEMF treatment has contributed to neural repair and protection of the dopaminergic neurons in the present study. The present study is the first to assess long-term treatment effect of T-PEMF and to our knowledge, the first to measure CSF levels of EPO in patients with PD.

As expected, the STS completion time at baseline was longer in the two PD groups than in the age-matched healthy reference group indicating bradykinesia. Our T-PEMF group (UPDRS-total: 46(SD 22), UPDRS-motor: 28(SD 15) medication in LED: 498 mg/day (SD 25)) performed 17% slower than the healthy reference group, while in a previous study a PD group (on-medication, UPDRS-total: 58(SD 18), UPDRS-motor: 35(SD 10), medication in LED: 758 mg/day (SD 412)) performed 35% slower than a healthy reference group [28, 35]. Despite the pharmacologically well medicated condition in our PD group, we found that the T-PEMF treatment intervention improved the completion time in the STS-task by 19% to a level comparable to the healthy reference group. Sub-division of the STS completion time into movement components indicated that the reduced STS completion time was explained by shorter times throughout the movement cycle.

We recently reported a positive effect of daily T-PEMF treatment for eight weeks on functional rapid force production in patients with mild PD. This may reflect an increased thalamo-cortical input to the motor cortex or increased excitability of the motor cortex, which might be due to higher levels of dopamine [15]. The mechanistic link between T-PEMF, CSF-EPO, and movement speed needs further investigation. However, as improved STS completion time after T-PEMF treatment was accompanied by increased CSF-EPO, we speculate on a potential connection between T-PEMF and cerebral levels of EPO, VEGF, and dopamine. EPO and VEGF have a common transcription factor, hypoxia inducible factor-1α (HIF-1α), which is upregulated in hypoxic conditions. At normal oxygen levels, HIF-1α is continuously and rapidly degraded. Oxygen-independent regulation of HIF-1α also occurs, however, and in normoxic conditions, increased endogenous nitric oxide seems to reduce degradation and thus stabilizes the HIF-1α level [36]. PEMF can induce nitric oxide mediated cerebral arteriolar dilation, leading to increased microvascular blood flow and tissue oxygenation in healthy rats [37]. It is thus plausible that our T-PEMF treatment up-regulated HIF-1α through increased production of nitric oxide, resulting in up-regulation of EPO and VEGF. HIF-1α also regulates tyrosine hydroxylase (TH), which is rate-limiting in dopamine synthesis [38]. TH levels are reduced in the striatum of post mortem PD brains and may contribute to the lower striatal dopamine production in patients with PD, although compensatory mechanisms seem to increase the activity of the remaining TH [39]. T-PEMF may thus improve functional performance by increasing the dopamine level in the brain mediated by EPO induced protection of dopaminergic neurons, via increased TH enzyme activity. However, this does not exclude additional influence of other growth factor mechanisms.

The present treatment effect on STS completion time was 3–4 times stronger after the longer treatment period (3x 8 weeks) than found in our previous study where the patients were treated for eight weeks only, possibly because this was sufficient time to induce structural changes in the brain while eight weeks were not [15]. We did apply to the Danish Medicines Agency for a longer treatment period before the eight-week study. However, at this point we were not allowed to treat the patients for longer than eight weeks due to lack of knowledge regarding adverse events. Our previous study showed no statistical difference between the treated group and the placebo group regarding reported adverse events. The present study supported this conclusion.

Furthermore, our previous study showed that an eight-week treatment period was beneficial for patients with mild PD whereas a significant treatment effect was lacking among the more severely affected participants [15]. However, the present study where the patients received the same treatment (30 min/day) but for 3x8 weeks showed a beneficial treatment effect for the entire study group including mild as well as more severely affected PD patients.

### Methodological considerations

Whereas pharmacological treatment of PD is associated with significant adverse effects, T-PEMF showed no significant adverse effects either over eight weeks [40] or over three periods of eight weeks as in the present study. T-PEMF was applied at home, and we found high compliance both in our previous eight-week study (97.9%) [16] and in the present 3x8-week study (97.6%). Home treatment is a major advantage as daily treatment in the clinic seems to be unrealistic. We observed unchanged plasma EPO concentrations despite increased CSF levels. This supports the results of animal studies showing that the blood brain barrier significantly limits the passage of EPO. Plasma concentration of EPO can thus not be used as a biomarker for CSF-EPO.

### Perspectives

The present study contributed with new knowledge regarding the effect of long-term T-PEMF treatment on movement speed and specific CSF biomarkers. To further understand the clinical perspective as well as the physiological mechanisms of action of the new treatment modality there is a need for knowledge on other outcome parameters such as e.g. Parkinson specific tremor, fine motor function and motor control, in future studies performed with the same T-PEMF intervention. Also, knowledge regarding duration of treatment effect in the follow-up period as well as the optimal treatment period would be valuable from a clinical perspective. Unfortunately, systematic investigation of the duration of treatment effect post treatment was beyond the scope of the present study, but a patient performed writing tests during and after the treatment period independently of the researchers. The patient’s handwriting ability improved significantly after the treatment period compared to baseline and this lasted approximately three months after treatment completion, indicating a possible long-term effect [41]. As the treatment effect is expected to level off over time, a longer treatment period, or perhaps life-long treatment may be considered for patients with PD. Finally, the effect of T-PEMF on other neurodegenerative diseases could also be relevant to study.

### Limitations

We included a PD-control group to allow comparison with the natural progression of the disease, but we did not have a PD-placebo group. As our previous study (RCT-trial) showed positive effects among patients with mild PD after eight-weeks of treatment, we decided for ethical reasons not to include a placebo PD group in the current study with longer treatment period, although this is a study limitation. However, since all outcome data were objectively measured, we do not expect this to be a major limitation.

A specific sample size calculation was not performed as previous data on the effect of long-term treatment did not exist. Our sample size considerations were based on previous experience with measurements of performance during the STS-task. The test-retest reliability of the STS-task in PD patients is substantial [42]. However, a limitation is that only a subgroup of the PD patients was included in the biomarker assessment, which increases the risk of type II error. Finally, the participants were recruited by convenience, largely based on consecutive recruitment from clinic (possible risk of allocation bias). Generalization should therefore be performed with caution.

## Conclusions

In patients with Parkinson’s disease, daily treatment with bipolar transcranial pulsed electromagnetic fields (T-PEMF) for three periods of eight weeks appeared to strengthen the natural protective/compensatory response in the human brain.

Motor performance in terms of movement speed was improved markedly compared to the natural progression seen in the PD-control group and CSF levels of erythropoietin increased in the T-PEMF group. Post treatment values of the STS completion time corresponded to the level of an age matched healthy reference group. These results suggest that T-PEMF has a neuro-restorative and/or a neuroprotective effect and that T-PEMF treatment is a potential innovative neuroprotective therapy in PD. However, the results do not exclude additional influence of other growth factor mechanisms.

## Supporting information

S1 Table STS completion time (s). (DOCX)

S2 Table STS completion time (% of baseline (week 0)). (DOCX)

S1 Data (XLSX)

S2 Data (XLSX)

S3 Data (XLSX)

S4 Data (XLSX)
