# Supplementary material for: Long-term treatment with transcranial pulsed electromagnetic fields improves movement speed and elevates cerebrospinal erythropoietin in Parkinson’s disease
Source: PLoS One. 2021 Apr 28;16(4):e0248800. doi: 10.1371/journal.pone.0248800 (PMC8081215; doi:10.1371/journal.pone.0248800)
Supplement: S1 Table — (DOCX) [file pone.0248800.s001.docx]

**S1 Table: STS completion time (s)**

| **Sit-to-stand completion time** | Baseline  Mean (SD)  (s) | Week 18  Mean (SD)  (s) | Week 27  Mean (SD)  (s) |
| --- | --- | --- | --- |
| T-PEMF group | 10.10(2.41) | 9.00(2.12) | 8.23(1.78) |
| PD control group | 9.41(1.06) | 9.48(0.92) | 9.63(0.94) |
| Healthy reference group | 8.64(1.61) | - | - |
