# Supplementary material for: Long-term treatment with transcranial pulsed electromagnetic fields improves movement speed and elevates cerebrospinal erythropoietin in Parkinson’s disease
Source: PLoS One. 2021 Apr 28;16(4):e0248800. doi: 10.1371/journal.pone.0248800 (PMC8081215; doi:10.1371/journal.pone.0248800)
Supplement: S2 Table — (DOCX) [file pone.0248800.s002.docx]

**S2 Table: STS completion time (% of baseline (week 0))**

| **Sit-to-stand completion time** | Baseline  Mean  (%) | Week 18  Mean  (%) | Week 27  Mean  (%) |
| --- | --- | --- | --- |
| T-PEMF group | 100 | 89.26 | 82.07 |
| PD control group | 100 | 101.07 | 102.86 |
